# Supplementary material for: Proprioceptive limit detectors contribute to sensorimotor control of the Drosophila leg
Source: Nat Commun. 2026 Feb 12;17:2664. doi: 10.1038/s41467-026-69333-z (PMC13009157; doi:10.1038/s41467-026-69333-z)
Supplement: Supplementary file 1 — Supplementary Information [file 41467_2026_69333_MOESM1_ESM.pdf]

Supplemental information

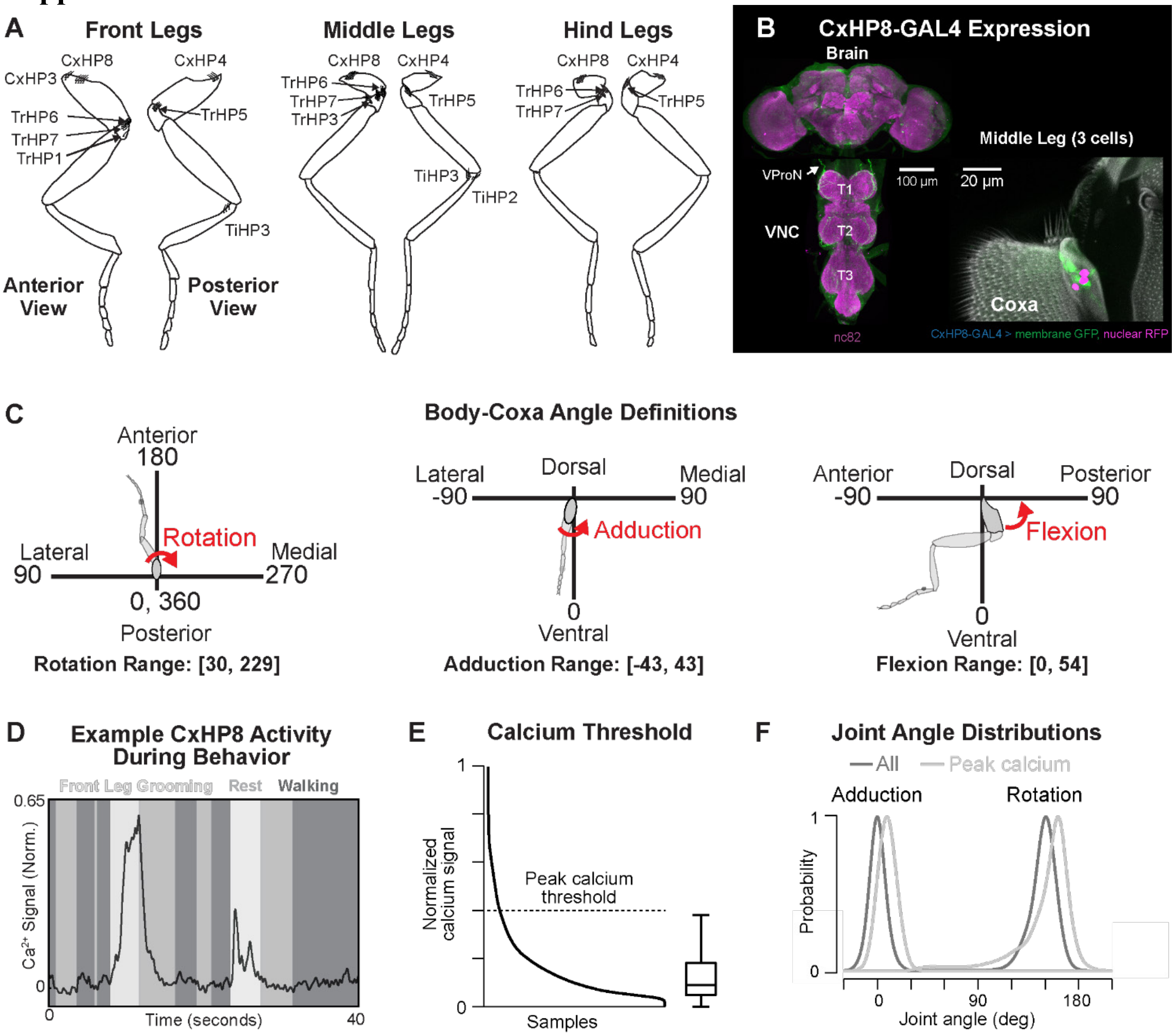

Figure S1. Hair plate locations on the legs of *Drosophila*, CxHP8 driver line expression, and thorax-coxa joint angle definitions and encoding. (**A**) Schematics of the front, middle, and hind legs showing the locations of hair plates. (**B**) Confocal images showing the expression of the CxHP8 split-GAL4 in the middle leg (3 of 8 neurons labeled), brain, and VNC. The ultrastructure (autofluorescence: gray) of CxHP8 consists of 8 cuticular hairs located on the coxa. Labeled mechanosensory neuron (GFP: green) project through the ventral prothoracic nerve (VProN) into the VNC (nc82: magenta). The cell bodies of CxHP8 neurons are labeled by the nuclear marker, redstinger (magenta). CxHP8 expression in the VNC and middle legs was imaged in 3 and 2 flies, respectively. (**C**) Reference frame for the rotation, adduction, and flexion thorax-coxa angles of the front leg. The measured range of each angle is displayed at the bottom. (**D**) Example trial showing the normalized calcium activity (normalized to the max calcium activity in the dataset) during front leg grooming, rest, and walking. (**E**) Calcium threshold was set at the upper whisker limit (Q3 + 1.5 * IQR) of the calcium activity box plot and was used for determining large peak calcium activity events. (**F**) Adduction and rotation joint angle probability distributions during all calcium activity (gray) and just peak calcium events (light gray).

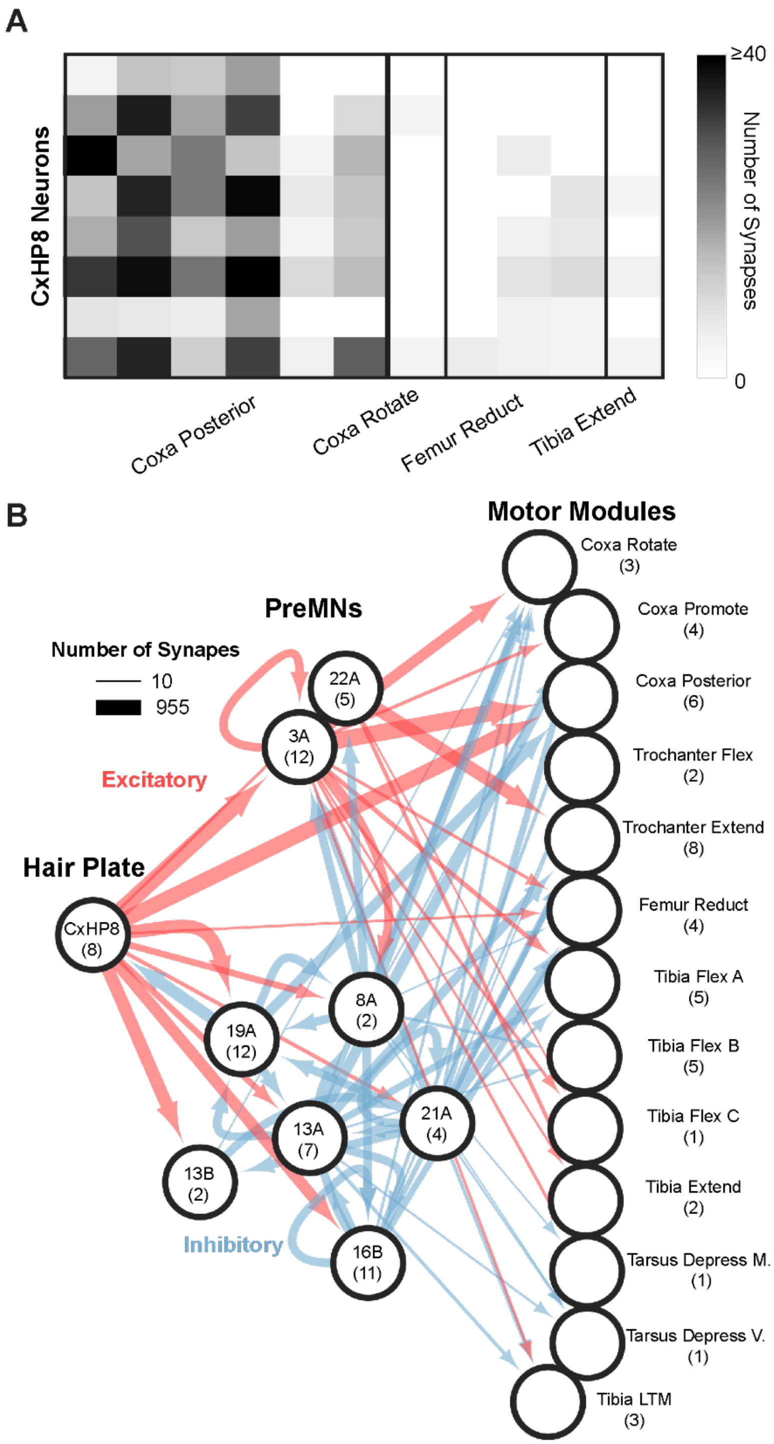

Figure S2. CxHP8 reflex circuit connectivity. (**A**) CxHP8 connectivity onto motor neurons reveals that all CxHP8 axons primarily synapse onto motor neurons within the coxa posterior motor module. The x-axis represents individual motor neurons within defined motor modules. (**B**) CxHP8 reflex circuit shown in **Figure 2B**, with the addition of premotor-to-premotor, and premotor-to-CxHP8 recurrent connections. Red and blue lines indicate excitatory and inhibitory connections, respectively. The width of the line corresponds to the number of synapses between cell classes. The number of neurons within each class are indicated within the parentheses. Note that only connections with greater than 3 synapses on average between upstream neurons within a class and downstream cell classes were used in this analysis.

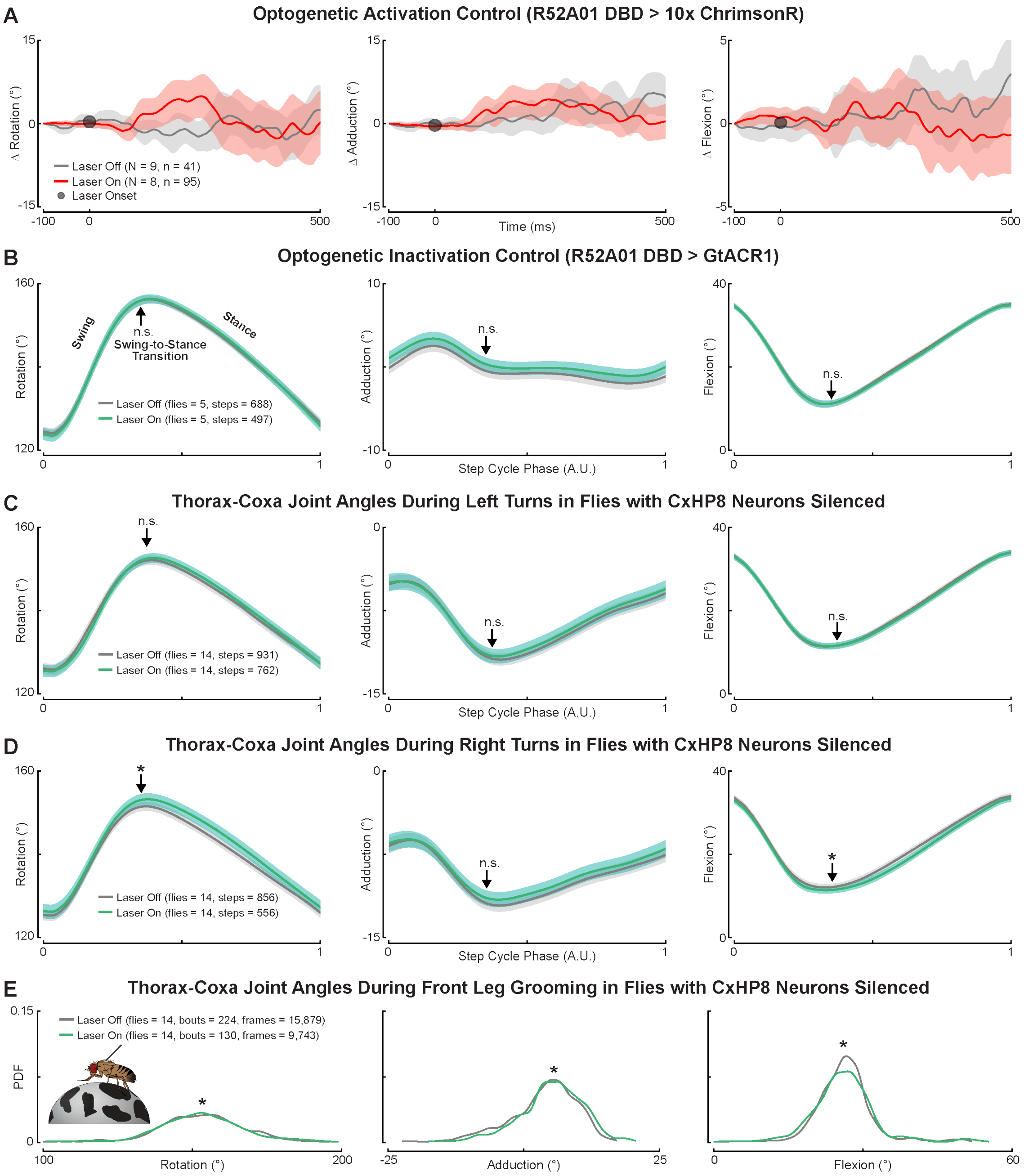

Figure S3. Control flies had no significant change in joint angles during laser stimulation and CxHP8 silencing had significant effects on thorax-coxa joint angles during right turns and front leg grooming but not left turns. (**A**) The red laser used in optogenetic activation experiments had a negligible effect on joint angles in control flies (R52A01 DBD > 10x ChrimsonR) compared to no laser trials. The joint angle trajectories are normalized to the start of the analysis window (i.e. 100 ms prior to laser). Red: laser on trials; Gray: laser off trials; Shaded region: 95% confidence interval of the mean; N: number of flies; n: number of trials. (**B**) Control flies (R52A01 DBD > GtACR1) had no significant (n.s.: $p > 0.05$) changes in coxa joint angles when the green laser was on compared to when it was off. Arrow: the average swing-to-stance transition of the step cycle; Shaded region: 95% confidence interval of the mean. A linear mixed-effects model was used to statistically compare the distributions of joint angles at the swing-to-stance transition. (**C**) Optogenetic silencing (green) of CxHP8 neurons in flies turning left (rotational velocity between -50 and -25 degrees/s) didn't have a significant effect (linear mixed-effects model; n.s.: $p > 0.05$) on thorax-coxa angles compared to turning steps when the laser was off. Shaded region: 95% confidence interval of the mean. (**D**) Optogenetic silencing of CxHP8 neurons had a significant (linear mixed-effects model; *: $p < 0.05$; Rotation: $p = 0.044$; Flexion: $p = 0.019$) effect on flexion and rotation angles at the swing-to-stance transition during right turns (rotational velocity between 25 and 50 degrees/s). Shaded region: 95% confidence interval of the mean. (**E**) Optogenetic silencing (green) of CxHP8 neurons significantly (linear mixed-effects model; *: $p < 0.05$; Rotation: $p = 9.185e^{-8}$; Adduction: $p = 5.88e^{-27}$; Flexion: $p = 2.43e^{-10}$) altered the thorax-coxa joint angle during front leg grooming compared to when the laser was off (gray). Shaded region: 95% confidence interval of the mean.

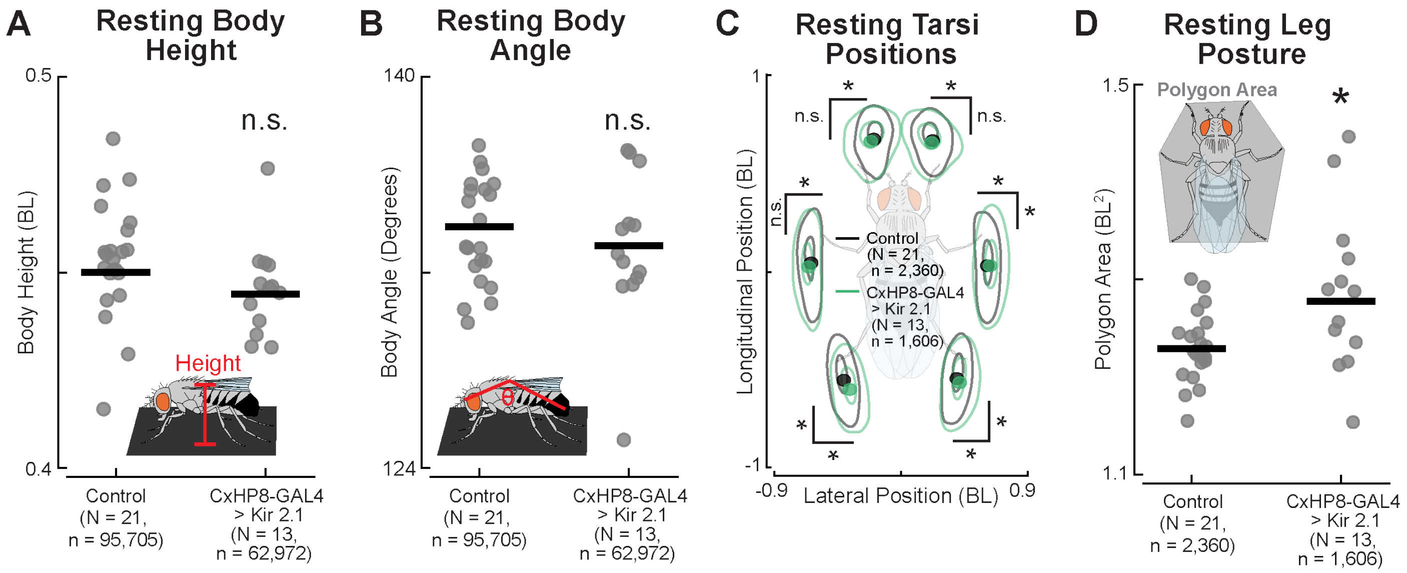

Figure S4. CxHP8 feedback controls resting leg positions but not body height or angle. (**A**) Body height was lower on average but not significantly different between CxHP8 silenced and control flies (two-sided t-test; $p > 0.05$). N: number of flies; n: number of frames. (**B**) Body angle was also smaller on average but not significantly different between CxHP8 silenced and control flies (two-sided t-test; $p > 0.05$). N: number of flies; n: number of frames. (**C**) Resting positions of the tarsi during periods of standing were significantly different between CxHP8 silenced (green) and control (black) flies. Distributions were determined by kernel density estimation. Dot: mean position; N: number of flies; n: number of resting bouts. A linear mixed-effects model was used to test for statistical differences (*: $p < 0.05$; L1 Lateral: $p = 0.018$; L2 Lateral: $p = 1.164e^{-17}$; L3 Lateral: $p = 1.565e^{-12}$; L3 Longitudinal: $p = 2.375e^{-13}$; R1 Lateral: $p = 2.5e^{-9}$; R2 Lateral: $p = 6.35e^{-32}$; R2 Longitudinal: $p = 3.75e^{-5}$; R3 Lateral: $p = 5.42e^{-57}$; R3 Longitudinal: $p = 7.098e^{-38}$). (**D**) As quantified by polygon area, the resting posture of the legs was significantly more spread out for flies with CxHP8 neurons silenced compared to controls (two-sided t-test; *: $p < 0.05$; $p = 0.0275$). N: number of flies; n: number of resting bouts.

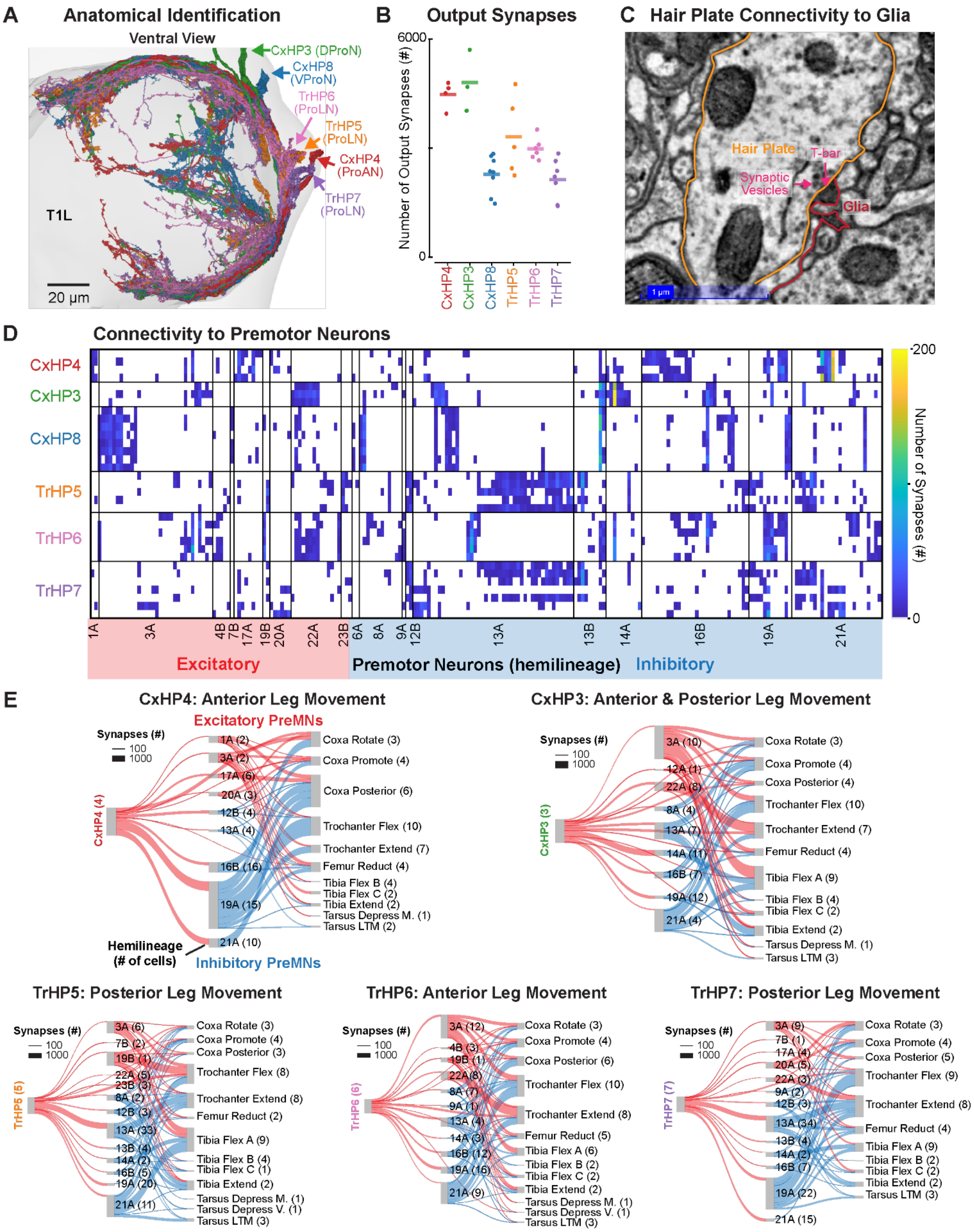

Figure S5. Synaptic connectivity of hair plates in motor circuits and onto glia. (**A**) Front leg hair plate axons are identified, in part, based on the nerve they project through and axonal morphology. DProN: dorsal prothoracic nerve; VProN: ventral prothoracic nerve; ProAN: prothoracic accessory nerve; ProLN: prothoracic leg nerve. (**B**) Total number of predicted output synapses of hair plate axons in FANC. Line: mean output synapses; dot: number of output synapses of a single hair plate axon. (**C**) Electron microscopy image illustrating the connectivity between a hair plate axon (orange) and a glia cell (red). Synaptic vesicles and a putative T-bar are labeled. (**D**) Connectivity matrix showing the number of synapses that hair plate axons supply to premotor neurons. Premotor neurons are classified as excitatory (red) or inhibitory (blue) based on their identified hemilineage. Premotor neurons within a hemilineage are arranged according to the proportion of input synapses they receive from each hair plate axon. (**E**) Based on their connectivity with premotor and motor neurons, the neurons of each hair plate are predicted to engage reflex circuits that control distinct aspects of leg movement. Above each reflex circuit is the prediction for the associated hair plate in controlling leg movement. Only connections of at least 4 synapses on average per neuron within a cell class onto downstream cell classes were used to construct each reflex circuit. The number of cells in each cell class is indicated within the parentheses.

Driver Line	Front Leg Proprioceptors										Middle Leg Proprioceptors							Hind Leg Proprioceptors					
	CxHP4	CxHP3	CxHP8	TrHP5	TrHP6	TrHP7	TrHP1	TrCS3 (TrG)	TrCS5 (TrFa)	FeCS11	CxHP4	CxHP8	TrHP5	TrHP6	TrHP7	TrCS3	TrCS2	CxHP4	CxHP8	TrHP5	TrHP6	TrHP7	TrCS3
R48A07 AD: R20C06 DBD	0	0	6	0	0	0	0	0	0	0	0	3	0	0	0	0	0	0	0	0	0	0	0
VT038115 AD: VT040055 DBD	0	0	0	2	0	3	0	0	0	0	N/A	N/A	N/A	N/A	N/A	N/A	N/A	N/A	N/A	N/A	N/A	N/A	N/A
VT0098323 AD: VT061711 DBD	1	0	0	2	0	2	0	2	0	0	N/A	N/A	N/A	N/A	N/A	N/A	N/A	N/A	N/A	N/A	N/A	N/A	N/A
VT0098323 AD: R20C06 DBD	0	0	0	0	0	2	0	3	0	1	N/A	N/A	N/A	N/A	N/A	N/A	N/A	N/A	N/A	N/A	N/A	N/A	N/A
R39B11 AD: VT061711 DBD	0	0	0	3	0	2	0	2	0	3	N/A	N/A	N/A	N/A	N/A	N/A	N/A	N/A	N/A	N/A	N/A	N/A	N/A
R48A07 AD: VT040055 DBD	0	0	0	1	0	1	1	0	0	0	N/A	N/A	N/A	N/A	N/A	N/A	N/A	N/A	N/A	N/A	N/A	N/A	N/A
VT038115 AD: VT061711 DBD	0	0	0	2	0	2	0	0	0	0	N/A	N/A	N/A	N/A	N/A	N/A	N/A	N/A	N/A	N/A	N/A	N/A	N/A
VT0098323 AD: R94E06 DBD	1	0	0	0	0	0	0	0	0	1	N/A	N/A	N/A	N/A	N/A	N/A	N/A	N/A	N/A	N/A	N/A	N/A	N/A
R22D12 AD: VT061711 DBD	2	0	0	0	1	1	0	3	2	3	N/A	N/A	N/A	N/A	N/A	N/A	N/A	N/A	N/A	N/A	N/A	N/A	N/A
R22D12 AD: R94E06 DBD	2	0	4	0	1	0	0	0	0	1	N/A	N/A	N/A	N/A	N/A	N/A	N/A	N/A	N/A	N/A	N/A	N/A	N/A
R48A07 AD: R22D12 DBD	4	0	0	0	3	0	0	0	0	0	N/A	N/A	N/A	N/A	N/A	N/A	N/A	N/A	N/A	N/A	N/A	N/A	N/A
R94E06 AD: VT040055 DBD	1	0	0	0	0	3	0	0	0	0	N/A	N/A	N/A	N/A	N/A	N/A	N/A	N/A	N/A	N/A	N/A	N/A	N/A

Table S1. Proprioceptor expression of split-GAL4 driver lines in the legs of *Drosophila melanogaster*. The number of cells labeled by the screened split-GAL4 driver lines was determined by counting cells genetically expressing membrane bound GFP and a nuclear reporter (i.e. UAS-mcD8GFP; Redstinger) using confocal imaging. Proprioceptors that did not have their expression checked received a “N/A”. The lack of a column for a particular proprioceptive class indicates that either no expression was seen or that proprioceptor was not checked for expression. HP: hair plate; CS: campaniform sensilla.
