# Supplementary material for: Proprioceptive limit detectors contribute to sensorimotor control of the Drosophila leg
Source: Nat Commun. 2026 Feb 12;17:2664. doi: 10.1038/s41467-026-69333-z (PMC13009157; doi:10.1038/s41467-026-69333-z)
Supplement: Supplementary file 2 — Description of Additional Supplementary Files [file 41467_2026_69333_MOESM2_ESM.pdf]

Description of Additional Supplementary Information Files:

Supplementary Video 1. Calcium activity of CxHP8 neurons while the left front leg was moved passively and slowly using a 3-axis platform.

Supplementary Video 2. Animation of hair plates on the front leg of a fly walking on a treadmill. The fly and hair plates were rendered in Blender and based on high resolution confocal images. Depicted are the three hair-plates located at the thorax-coxa joint (CxHP4: red; CxHP3: green; CHP8: blue) and three at the coxa-trochanter joint (TrHP5: orange; TrHP7: purple; TrHP6: pink).

Supplementary Video 3. Calcium activity of CxHP8 neurons while a fly behaved on a spherical treadmill.

Supplementary Video 4. Optogenetic activation of CxHP8 neurons drives posterior leg movement in standing flies. The video is slowed by 5x. The example trial was 2 seconds, and the laser was presented for 1 second (red square) after 0.5s from the start of the trial.
